# The Widespread Use of Nanomaterials: The Effects on the Function and Diversity of Environmental Microbial Communities

**DOI:** 10.3390/microorganisms10102080

**Published:** 2022-10-21

**Authors:** Chunshui Sun, Ke Hu, Dashuai Mu, Zhijun Wang, Xiuxia Yu

**Affiliations:** 1 College of Marine Science, Shandong University, Weihai 264209, China; 2 Institute for Advanced Study, Chengdu University, 2025 Chengluo Avenue, Chengdu 610106, China

**Keywords:** Nanomaterials, ecotoxicology, environment microbial communities

## Abstract

In recent years, as an emerging material, nanomaterials have rapidly expanded from laboratories to large-scale industrial productions. Along with people’s productive activities, these nanomaterials can enter the natural environment of soil, water and atmosphere through various ways. At present, a large number of reports have proved that nanomaterials have certain toxic effects on bacteria, algae, plants, invertebrates, mammalian cell lines and mammals in these environments, but people still know little about the ecotoxicology of nanomaterials. Most relevant studies focus on the responses of model strains to nanomaterials in pure culture conditions, but these results do not fully represent the response of microbial communities to nanomaterials in natural environments. Over the years, the effect of nanomaterials infiltrated into the natural environment on the microbial communities has become a popular topic in the field of nano-ecological environment research. It was found that under different environmental conditions, nanomaterials have various effects on the microbial communities. The medium; the coexisting pollutants in the environment and the structure, particle size and surface modification of nanomaterials may cause changes in the structure and function of microbial communities. This paper systematically summarizes the impacts of different nanomaterials on microbial communities in various environments, which can provide a reference for us to evaluate the impacts of nanomaterials released into the environment on the microecology and has certain guiding significance for strengthening the emission control of nanomaterials pollutants.

## 1. Introduction

**Figure 1:**
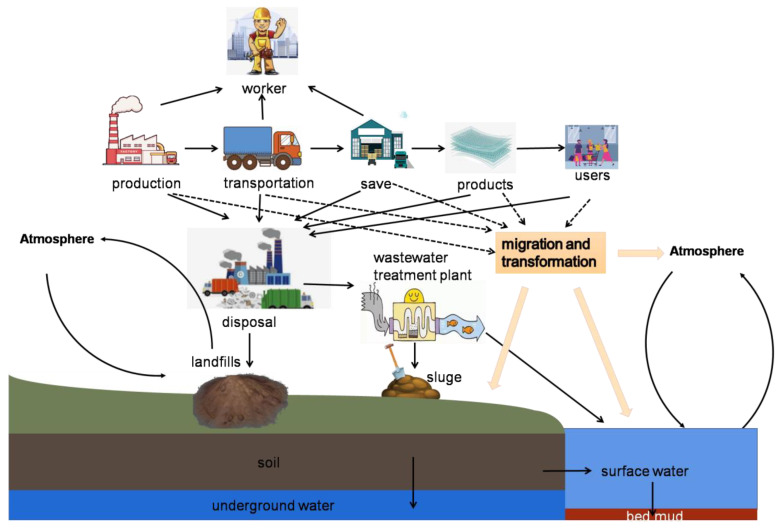
Routes of migration and transformation of NPs in the environment.

The concept of “Nanotechnology” was first proposed by Nobel Laureate Richard P. Feynman in his famous lecture “There’s Plenty of Room at the Bottom” in 1959. In 1990, the first International Nanoscience Conference was held in Baltimore, United States, which marked the birth of nanoscience as a new branch of material science. Nanomaterials are known as “the most promising materials in the 21st century” because the basic unit size of nanomaterials is small. Compared with conventional materials, nanoscale materials exhibit unique physical and chemical properties, such as surface effect, small size effect, quantum size effect and macroscopic quantum tunneling effect. This makes nanoparticles (NPs) have special optical, electrical, magnetic, mechanical, superconductivity and catalytic properties and show broad application prospects in promoting social development and improving human life. With the rapid development of nanotechnology, a large number of consumer goods containing NPs and nanoparticle products have been widely used in various fields such as jewelry, photography, cosmetics, aquaculture, agriculture, medicine, textiles, energy electronics and aerospace industry [1,2,3,4,5,6,7,8,9,10,11]. However, nanomaterials are also a double-edged sword. They accelerate the development and innovation of industry and play an important role in promoting social development and progress but also bring some negative effects. In the process of their production, use, transportation and disposal, nanomaterials will inevitably enter the environments of soil, water and atmosphere through various ways. Similar to other pollutants, these NPs will reenter the natural environment and participate in the material cycle through migration and transformation in soil, water, atmosphere and organisms in these environments [12] (Figure 1). In this process, NPs interact with the organisms and are absorbed and enriched, directly or indirectly threatening the ecosystem and human health [13,14]. Many studies have been reported on the toxicity of NPs to bacteria, algae, plants, invertebrates, vertebrates, mammalian cells and mammals [15,16,17,18,19,20,21,22,23,24,25,26,27,28], and these studies also confirm that NPs have potential environmental hazards. Although there are many reports on the effects of nanomaterials on single species of organisms, the research on nano ecotoxicology is still in its infancy [29].

Among these subjects, microorganisms, as one of the most biodiverse species on earth, are the basis of the entire food chain and play a leading role in the earth’s material and energy cycles. Because microbial communities have these important ecological functions and their ubiquitous distribution characteristics in environmental media, it is particularly important to study the eco-toxicity of NPs to microbial communities. Studies have shown that NPs in soil, water and atmosphere can affect the growth and physiological activity of microorganisms by coexisting with microorganisms, adsorbing on the surface of microorganisms and even entering the organism. However, the impact of NPs on the structure and diversity of microbial communities is much more complicated. The type, exposure time and concentration of NPs will have different effects on the structure and diversity of microbial communities [30,31]. Similarly, the heterogeneity of different environments brings about different environmental changes to NPs, leading to different effects of the same type of NPs on the diversity of microbial communities [32]. In addition, some NPs have been found to have certain effects on specific bacterial groups. Therefore, the reasonable evaluation of the harm of NPs to the structure and diversity of microbial communities is of great significance to the scientific development of nanotechnology and the safe use of NPs.

## 2. Effects of NPs on Microbial Community in Aquatic Environment

In recent decades, with the rapid development of nanotechnology, many consumer products containing NPs and nanoparticle products have been widely used in all aspects of life. For example, coatings containing titanium dioxide NPs can improve the ultraviolet reflectivity of building materials, increasing the ability of anti-aging and pollution resistance. However, the NPs in the coatings are easily released into the ecosystem after being washed by rain [33]. Fabrics containing nano silver (Ag NPs) will release nano silver into the water environment during cleaning [34]. Therefore, the wide application of nanomaterials makes NPs a potential pollution in the water environment. These nanomaterials may eventually enter the wastewater treatment system with domestic sewage or industrial wastewater. At the same time, nanomaterials with excellent surface chemical properties can be used as adsorbents and flocculants in the wastewater treatment process [35,36,37,38]. These applications also increase the number of NPs in wastewater treatment plants to a certain extent. In addition to the point source approach mentioned above, nanomaterials can also enter the water environment through non-point sources [39]. The nanomaterials in the soil can penetrate downwards and eventually cause groundwater pollution, or after being washed by rainwater, some of the NPs will flow into natural water bodies such as rivers, lakes and oceans along with surface runoff [40]. The NPs in the atmospheric environment will also return to the ground and enter the water environment in the form of wet sedimentation.

The aquatic environment is a focus of research on the environmental behavior of nanomaterials because it is the main medium through which nanomaterials enter and diffuse into other environmental resources, playing a “link” role [41]. The investigation of the effects of nanomaterial exposure on the aquatic environment is very important because the aquatic environment receives runoff and wastewater from domestic and industrial sources, and is an important gathering place for various pollutants [42]. In this section, we will discuss in detail the effects of nanomaterials on microbial community structures in natural water bodies such as rivers, lakes and oceans, as well as in unnatural water bodies such as wastewater treatment plants.

### 2.1. Effects of NPs on Microbial Community in Wastewater Treatment Plants

As a new pollutant, NPs are likely to be discharged into wastewater treatment systems in the process of production, use and disposal [43,44]. At present, wastewater treatment plants mainly adopt activated sludge technology, and microorganisms play a leading role. The activity, composition and structure of microbial communities determine the treatment efficiency of wastewater treatment plants to a certain extent. The entry of nanomaterials may affect the community structure, abundance and functions of microorganisms in activated sludge, thereby affecting the biological treatment effect of sewage, which has gradually attracted the attention of researchers. Studies have shown that depending on the type of NPs, exposure concentration, exposure time, surface modification and type of wastewater treatment process, NPs will have varying degrees of impact on the wastewater treatment system. A certain concentration of NPs will affect the microbial community in activated sludge, including the impact on the number of microorganisms and the change of diversity.

Ag NPs is widely used and has excellent antibacterial properties. Therefore, it is of great significance to study the potential impact of Ag NPs on the structure of microbial community in wastewater treatment systems. Button et al. [45] demonstrated that at low doses, Ag in either ionic or NP form did not produce significant toxic effects in the short term, but did lead to subtle changes in the function and structure of the microbial community. At higher doses, Ag NPs significantly altered the function of microbial communities. Moreover, different coatings on the surface of Ag NPs showed different toxicity. Xu’s group [46] evaluated the changes in performance, microbial community and enzyme activity of a sequencing batch reactor (SBR) in the presence of Ag NPs. The results show that a certain concentration of Ag NPs will inhibit the chemical oxygen demand (COD) and phosphorus removal efficiency of the reactor. Due to the response of microorganisms to the toxicity of Ag NPs, the release of reactive oxygen species (ROS) and lactate dehydrogenase (LDH) in the system increased, and the richness and diversity of microorganisms also changed significantly. Similarly, in a SBR, compared with the microbial community exposed to silver ions (Ag^+^), the change in and recovery speed of the microbial community exposed to Ag NPs is slower, and the recovery speed of the microbial community is also different due to the different coating on the surface of Ag NPs [47]. When Ag NPs were added to a vertical flow constructed wetland (VFCW) [48], the removal efficiency of organic matter did not change significantly, but the removal efficiency of nitrogen and phosphorus had a certain effect. High-throughput sequencing results showed that the presence of Ag NPs changed the relative abundance of functional bacteria associated with nitrogen and phosphorus removal.

As one of the three most widely used inorganic nanomaterials in commercial products [49], nano zinc oxide (ZnO NPs) has a large industrial production, a wide range of applications and a relatively large potential risk of discharge into the environment [50]. Hu’s study [51] showed that high concentration of ZnO NPs would lead to a decrease in microbial community richness in the system. Wu et al. [52] discussed the impacts of zinc (Zn) NPs, ZnO NPs and Zn ions (Zn^2+^) on the nitrifying bacterial communities. When zinc concentration is low, Zn^2+^ can improve the nitrification rate, but at high concentrations, both zinc NPs inhibit the nitrification rate. This result suggests that it is the NPs themselves, rather than the released Zn^2+^, that are responsible for the toxicity to nitrifying bacteria. The authors suggest that the presence of NPs can cause nitrifying bacteria to produce large amounts of ROS. However, Zhang’s work [53] shows that under the condition of low concentration, the existence of ZnO NPs is conducive to the removal of nitrogen, and the inhibition effect can be produced only at high concentration. Long-term exposure to ZnO NPs will lead to declines in microbial diversity. Chen’s group [54] investigated the effects of four conductive nanomaterials (carbon powder NPs, Al_2_O_3_ NPs, ZnO NPs, CuO NPs) on sludge anaerobic digestion (AD) performance and microbial community. The experimental data showed that carbon powder NPs and Al_2_O_3_ NPs could improve the biogas production of AD, while ZnO NPs and CuO NPs had the opposite effect, which was caused by the fact that ZnO NPs and CuO NPs reduced the diversity and richness of microbial community. The toxic effects of CuO, ZnO and TiO_2_ NPs on the nitrogen removal, microbial activity and community were also compared by Zhang’s group [55]. The results showed that all three NPs were toxic to the Anammox process, leading to a decrease in nitrogen removal efficiency and microbial activity. The comparison results showed that the toxic effect of CuO NPs was the most serious, but the cumulative effect was the least, while the toxic effect of TiO_2_ NPs was the least, but the cumulative effect was the most serious. This result also indicates that different types of NPs will have different effects on the same system.

Nano zero valent iron (nZVI) is one of the most widely used nanomaterials in the remediation of polluted soil and groundwater. It mainly aims at the biodegradation of chlorinated compounds, the fixation of heavy metals and the adsorption of inorganic anions in the field of pollution control [35,56,57,58,59]. In addition, maghemite (γ-Fe_2_O_3_), hematite (α-Fe_2_O_3_) and magnetite (Fe_3_O_4_) are also the most common magnetic nanomaterials applied in remediation and water treatments. Many studies have shown that nZVI and magnetic NPs can affect biochemical processes in microorganisms and microbial communities in the activated sludge of sewage treatment plants. The effects of nZVI on sludge anaerobic digestion were investigated by Yu’s group [60]. Data showed that the initial addition of nZVI led to a decrease in methane production. However, with the adjustment of microbial community structure to adapt to environmental changes, the yield of methane increased, indicating that nZVI could directly affect sludge fermentation liquor and microbial community structure. The same results were also obtained in Pan’s research [61]. The results showed that the presence of nZVI could improve the abundance of methanogenic bacteria and promoted the production of methane. Magnetic Fe_3_O_4_ NPs have been proved to have little effect on the removal of NH_4_^+^-N and a slight effect on the removal of COD. The production of ROS and the release of LDH increased with the increase of magnetic Fe_3_O_4_ NPs in the system, indicating that magnetic Fe_3_O_4_ NPs has toxicity to activated sludge. High-throughput sequencing results also confirmed that magnetic Fe_3_O_4_ NPs did change the structure and diversity of microbial communities in the system [62]. Zhang’s work [63] pointed out that the existence of high concentration of maghemite NPs would not affect the anammox activity, the production of ROS or the integrity of cell membrane, and the long-term addition of high concentration of maghemite NPs had no adverse effect on the performance of the high-rate anammox reactor. On the contrary, with the increase of maghemite NPs concentration, the anammox activity increased. The experimental results showed that maghemite NPs had good biocompatibility and could be used to improve the characteristics of anammox flocculent sludge.

The effects of other nanomaterials on microbial community in sewage treatment system have also been reported. The effects of TiO_2_ NPs on the performance and microbial community of activated sludge in a SBR were studied by Li’s group [64] and Zheng’s group [65], respectively. The research results of the two groups showed that TiO_2_ NPs had certain toxicity to the microbial community and significantly changed the richness and diversity of the microbial community, resulting in the degradation of activated sludge performance. Wang’s group [66] proved that the presence of CeO_2_ NPs had obvious effect on the microbial richness and diversity of activated sludge, and his another work [67] also confirmed that nickel dioxide NPs (NiO NPs) had a similar impact on sludge microbial communities. In addition, a series of works has also proved that Bi_2_WO_6_ NPs, silica NPs and copper oxide NPs had varying degrees of influence on the microbial community in sewage treatment system [68,69,70].

The ecotoxicity of nanomaterials is affected by many factors, which is not only related to their own physical and chemical properties but also has a variety of different interaction relationships with activated sludge, natural particles, organic pollutants and biological macromolecules in the sewage treatment system [71], increasing the difficulty and complexity of the ecotoxicity research of nanomaterials. There is still no general consensus on the law and mechanism of the influence of nanomaterials on the wastewater treatment process and the impact on the environment. 

### 2.2. Effects of NPs on Microbial Community in Natural Water Bodies

With the development of agriculture, industry and urbanization, human pollution of water resources is becoming more and more serious. Estuaries and offshore areas are important transitional areas where oceans and rivers or land meet. This area plays a vital role in regulating the geochemical cycle of materials, human health and sustainable development [72,73]. Microorganisms in estuaries and marine sediments play an irreplaceable role in pollutant transformation, nutrient cycling and maintaining ecosystem health [74,75,76]. It has important ecological value and has always been hot spots in environmental geoscience research.

It has been found that the microbial community changes significantly when the natural river water is exposed to the environmental concentration of ZnO NPs [77]. In addition, studies have shown that the impacts of Ag NPs of different sizes and coatings on freshwater sediment microbial community may be significantly influenced by the conditions [78]. Du’s group [79] evaluated the effects of ZnO NPs on ecosystem function by studying the decomposition of leaf litter by microbial communities in aquatic ecosystem. The experimental data showed that ZnO NPs could significantly reduce the degradation rate of leaf litter in freshwater system. However, the transformation process (aggregation, settlement and dissolution) of ZnO NPs with various diameters in natural water was nother, resulting in different effects on microbial communities in water body. In addition, studies have assessed the effects of AgNPs on microbial communities that decompose leaf litter in river ecosystems [80]. Evidence showed that the microbial community structure changed during short-term exposure to AgNPs, but the metabolic activities of microorganisms were not affected. However, when Ag NPs were exposed for a long time, the structure and metabolic function of microbial community were strongly affected. Another study assessed the effects of coated and uncoated Ag NPs on oxygen consumption in freshwater benthic microbial communities [81]. The experimental results showed that the presence of coating on the surface of Ag NPs had a significant effect on their ecotoxicity. Uncoated Ag NPs were more ecotoxic. In another work [82], the authors evaluated the effects of three different types of nano-sized polystyrene (nPS) on the structure and function of freshwater microbial community. The results showed they were less ecotoxic than expected and basically had no effect on the function of the microbial community.

The ocean is one of the largest ecosystems in the world, and just like that, the ocean has become the largest recipient of pollutants. Marine microorganisms need not only to deal with the adverse effects of marine climate change but also to respond appropriately to the environmental pollutants in the ocean. Due to the complexity of the marine environment and the diversity of microbial composition, there are few reports on how NPs affect the marine microbial community.

Biogenic palladium NPs (bio-Pd NPs) can degrade or transform heavy metals, pesticides and organic halides in water, air, soil and sediments through catalysis, so they are widely used in the remediation of polluted environments [83]. The toxicity of bio-Pd NPs to a marine microbial community was evaluated by Nuzzo’s team [84]. Some respiratory metabolic effects of microorganisms were slightly inhibited by bio-Pd NPs, and the diversity of microbial community was slightly increased, which was the adjustment of microorganisms to cope with the toxicity of bio-Pd NPs. Overall, bio-Pd NPs in the study had little effect on the marine microbial community. Another work compared the effects of polymer-coated Ag NPs and Ag^+^ on marine microbial communities [85]. Compared with the control seawater, the microbial community richness in the seawater treated with polymer-coated Ag NPs and Ag^+^ was affected, although there was a long lag phase, indicating that some bacteria were not sensitive to polymer-coated Ag NPs and Ag^+^ or can adapt to the existence of polymer-coated Ag NPs and Ag^+^ in the environment. 

## 3. Effects of NPs on Soil Microbial Community

In recent years, nanomaterials have been incorporated into plant nutrition and disease management as fungicides and nano fertilizers [86,87,88]; among them nano pesticides and nano fertilizers are the most widely used agricultural products. In addition, some nanomaterials have been used in the remediation of contaminated soil [89]. As a result, the large-scale use of nanomaterials leads to increasing opportunities for and quantities of nanomaterials leaking into the soil directly. In addition to directly entering the soil through the above means, nanomaterials can also be collected from water and air into the soil through precipitation, atmospheric deposition and irrigation [90,91,92]. Furthermore, due to the weak migration ability of nanomaterials, soil will eventually be the main final recipient of nanomaterials in the environment compared with water and air [14,93,94,95,96].

With genetic diversity, functional diversity and community diversity, soil microorganisms participate in almost all biochemical reactions in the soil and are closely related to the quality of the soil, the ecosystem and the growth and productivity of plants [97]. In recent years, it has been reported that the content of organic matter and humic substances in soil is the main factor affecting the adsorption of NPs in soil [98,99,100,101], and these substances are important nutrient sources of soil microorganisms. Therefore, once NPs enter the soil environment, the impact on microbial community is inevitable. Soil has very complex and diverse special properties, such as pH, organic matter, ionic properties, mineral composition, particle size distribution and complex pore structure [90,99,101,102]. These factors will affect the migration and transformation behavior of NPs in soil [99,103], resulting in different disturbances to the abundance and community composition of soil microorganisms.

### 3.1. Effect of Nanomaterials on Microbial Community Structure and Diversity

Studies have reported that the type of nanomaterials as well as the soil types can affect the activity and the community of soil microorganisms differently [15]. Currently, multiple studies have initially revealed the impact of nanomaterials (metal and nonmetal NPs) on microbial communities under different conditions with specific nanomaterials, soil type, exposure time and concentration.

Studies have confirmed that heavy metal pollution in soil impairs microbial community structure and diversity. The different species and characteristics of metal NPs will have different effects on the structure and diversity of soil microbial community. Commercial Ag NPs are increasingly used in a variety of consumer products, which greatly increases the risk of their environmental release and soil accumulation. Of all the reports existing so far, Liu’s results [104] showed that Ag NPs will have a short-term impact on the structure and diversity of soil microbial community, but the microbial community will return to normal level after long-term exposure. On the contrary, another study [105] found that the effects of Ag NPs on microbial communities were long-lasting, and the longer Ag NPs existed, the more significant the effects became. The different results can be attributed to the heterogeneity of the different environments, which brings about different environmental responses to the NPs, leading to the different effects of the same NPs on microbial community diversity. Metal NPs are also affected by multiple factors in soil and affect the microorganisms in a dynamic and long-term process. Therefore, assessing the ecological risk of soil microbial communities to metal NPs should trace their environmental behavior in soil over a long time. Wang’s work [106] suggested that Ag NPs could have an effect on microbial communities, and the effect depended on the dose of Ag NPs. In addition, another work [107] assessed the different effects of Ag NPs on microbial communities in terms of particle state, ion release and shape. The results showed that the relative contribution of particle of Ag NPs to toxicity increased with increasing Ag NP concentration, and the toxicity of Ag NPs to the microbial community was different with different shapes. At the same time, the authors also emphasized that the results obtained in a relatively simple laboratory environment cannot fully reflect the real situation of Ag NPs in the natural environment, which should be noted by all researchers. Kumar et al. studied on the influence of NPs (Ag, Cu) on soil in the possibly vulnerable ecosystems of polar region. As a result, Cu NPs were not found significant effect on polar soil bacteria, but Ag nanomaterials showed highly toxic to these arctic consortia [108].

Researchers are studying multiple kinds of metallic oxide nanomaterials for their effects on microbial communities. In Ge’s work [31], the group investigated the effects of TiO_2_ and ZnO NPs on natural soil bacterial communities. The results showed that although the two kinds of NPs had certain toxicity and changed the structure and diversity of soil microbial community, their dose–response curve and the structural changes in the microbial community were different, indicating that the ecological toxicity of different NPs was affected by other factors such as composition, size and shape. In addition to the direct toxicity of NPs, the authors suggest that NPs may alter soil properties to indirectly affect microbial community structure, or environmental factors of soil may mediate the effects of NPs on microbial community. The authors also confirmed through experiments that the effect of TiO_2_ on soil microbial community was indeed mediated by soil water [109]. Researchers in 2021 [110] came to a similar conclusion that TiO_2_ NPs could affect the biomass of microorganisms in clay soil, but in another study [111] using metal NPs to repair agricultural soil, the type and dose of NPs used affected the soil microbial community to varying degrees, depending on the type, concentration and dissolution behavior of NPs. The results of this study showed that the high dose of TiO_2_ NPs did not affect the structure of the soil microbial community. Hankui Chai [112] exposed agricultural soil to ZnO, SiO_2_, TiO_2_ and CeO_2_ NPs and found that ZnO and CeO_2_ NPs significant inhibited numbers of soil *Azotobacter*, *P-solubilizing* and *K-solubilizing* bacteria, TiO_2_ NPs reduced the abundance of functional bacteria, and SiO_2_ NPs slightly boosted the soil microbial activity.

Nonmetal NPs such as graphene oxide (GO) [113] and carbon nanotubes (CNTs) were also studied about the effect on the microbial communities in soil. Haegeun Chung [113] treated soil with GO and found that GO lowered the soil enzyme activity in short term and had no significant effect on microbial biomass. Fei He [114] incubated farmland soil repeatedly treated with different concentrations of CNTs and found that different CNT doses and exposure times affected enzyme activity significantly differently and indicated that the repeated addition of CNTs affected the structure and function of soil microorganism communities. However, in some studies, it was found that some kinds of nanomaterials hardly affect the microbial communities in soil significantly, such as Pd, Au, C60, Al_2_O_3_, SiO_2_ and Cu [115,116,117,118,119,120].

### 3.2. Response of Typical Microbial Groups to NPs in Soil

Typical microorganisms are sensitive or slow to external environmental changes in many microbial species, and they are important or have special functions in the soil ecosystem. Microbial taxa with organic matter decomposition have certain tolerance to NPs in the external environment. When exposed to pollution, they can improve the influence of microbial community for more energy intake from the external environment to adapt to the adverse living conditions, and thus affect the material transformation in the soil circulation. In Ge’s study [31], abundances of *Streptomyce, Streptomycetaceae* and *Sphingomonadaceae* showed significant positive correlations with the concentration of ZnO and TiO_2_ NPs. He’s study [121] showed that the relative abundance of *Nocardioides*, *Actinobacteria*, *Streptomycetaceae* and *Duganella*, which are all involved in the decomposition of organic matter in soil increased significantly when they were exposed to the Fe_3_O_4_ NPs. Shrestha [119] found that multi-walled carbon nanotubes (MWNTs) could promote the growth of *Cellulomonas*, *Pseudomonas*, *Rhodococcus* and *Nocardioides*, and inhibit *Holophaga*, *Waddlia*, *Derxia* and *Opitutus.* The tolerance of these bacterial under the nanomaterials stress enables them to survive and reproduce. This survival advantage in the acute exposure may have a relatively large survival probability with high concentration of nanomaterials.

However, the relative abundance of increased or decreased microbial communities does not necessarily mean the increase or decrease of the absolute content of the taxa. It is highly likely that the exposure of microorganisms to NPs causes the decline of their total amount, while some resistant bacteria become dominant taxa. Therefore, in the process of studying microbial typical taxa, a variety of related biological biochemical and physiological indicators should be combined to better understand the response characteristics and mechanisms of typical microbial taxa to nanoparticle stress.

## 4. Discussion on the Effects of NPs on Microbial Communities in Different Environments

In Table 1, we summarize the recent knowledge about the impacts of NPs on the structure and function of microbial communities in different environments and provide a reference for us to evaluate whether the release of nanomaterials into the environment may pose a potential risk to the environmental microbial communities.

To investigate the ecological effect and mechanism of nanomaterials on microorganisms in activated sludge, we should not only detect the changes in individual levels of microorganisms, such as microbial morphological changes and cell survival status, but also explore the succession of microbial community and the changes in functional genes. Researchers should continue to explore to better predict the potential role of nanomaterials in wastewater biological treatment systems and to find more effective ways to mitigate adverse effects or make full use of the strengthening effect of nanomaterials on the performance of wastewater biological treatment system to improve the efficiency of wastewater treatment.

Compared with other environmental systems, natural water bodies accept a large amount of various materials from other environmental inputs in a typically complex interface of atmosphere, water and sediment, and the gradients of various environmental factors (physical, chemical, biological and other factors) change dramatically. In this complex environment, it is particularly difficult to study the effect of nanomaterials on microbial communities because it is difficult to determine whether changes in the microbial community are caused by the nanomaterials themselves or by the environment, or even nanomaterials interacting with other chemical substances in the environment.

Due to the high heterogeneity of soil, the limitation of orthotopic tracking technology and microbiological detection methods, the effect mechanism of NPs on the structure and diversity of microbial community still needs to be deeply studied.

On the whole, the current studies on nanomaterials are mainly focused on their short-term effects, while there are few studies on their long-term effects, and the research on microbial communities is not very in-depth; further research and discussions are needed. Studies on the ecological toxicity of nanomaterials are mainly carried out in a single laboratory environment, which cannot accurately reflect the ecological effects of nanomaterials in the real environment. The toxicity mechanism of nanomaterials mainly focuses on their toxicity to a single microorganism, and there are very few studies on their ecotoxicity mechanism, so it is hard to make an accurate and specific evaluation.

Furthermore, the study of nanomaterial toxicity in the environment is difficult and complex due to the multiple relationships between NPs and microorganisms and between NPs and the environment, all of which have been shown to alter nanomaterial toxicity in model systems. It is not difficult to see from our summary of existing studies that there are situations where the research results of different teams are contradictory. The occurrence of this situation also indicates that there are different influencing factors in different ecological environments, which leads to different ecotoxicity of nanomaterials [108].

In addition, combined pollution is also a problem that cannot be ignored in studying the effects of nanomaterials on microbial communities. Combined pollution refers to the environmental pollution by multiple pollutants with different properties (or different sources of the same pollutant) simultaneously existing in the same environmental medium [122]. It is a common pattern that chemical pollutants exist in various mixed forms in the environment [123]. The combined exposure of multiple chemical pollutants may produce stronger synergistic effects than single exposure, or weaker antagonistic effect than single exposure, or equivalent to the additive effect of single exposure. The basic principles of toxicology can be used to predict the health risks of a single chemical contaminant, but it is difficult to accurately predict the risks of mixing two or more chemical substances. If nanomaterials are present in the composite system, the combined effects of various chemical pollutants in the mixed system will be more difficult to predict because nanomaterials not only have unique physical and chemical properties but also often interact with other chemicals in various forms and mechanisms. Therefore, when studying the biological effects of nanomaterials, we should not only consider the toxic effects of individual NPs but also consider the interactions between NPs and other chemical pollutants [124]. It has been found that when organisms are exposed to multiple chemical pollutants simultaneously, the effects are significantly different from those produced by a single pollutant [125,126].

## 5. Conclusions

In the future, we should strengthen the study of the toxicity of nanomaterials in real environments, further explain the ecotoxicity mechanisms of nanomaterials and introduce new molecular technologies to improve the research system on the ecological effects of nanomaterials and accelerate the research process from nano ecotoxicology to nano ecotoxicology genomics. Our results should be interpreted with caution, as it remains uncertain whether the patterns observed in the laboratory incubation of the current study reflect those occurring in natural systems.

In the study of the combined toxicity of mixed chemical pollutants, evaluating the mode of action of combined toxicity of chemical pollutants is one of the important research tasks. It is of great significance to study the combined toxic effects of mixed NPs for their possible environmental risk assessment. Evaluation and prediction of mixed toxic effects of pollutants in the environment is one of the research hotspots in the field of ecotoxicology.

Nanomaterials are widely used and enter the environment, which brings potential risks to environmental ecology, and their toxic effects on organisms may affect the balance of the entire ecosystem. Ecotoxicity studies on nanomaterials mainly focus on the individual toxicity studies of nanomaterials on ecological species, while the joint toxicity studies on multicomponent nanomaterials on ecological species are still very limited, especially for the joint toxicity between nanomaterials with different dimensions and the combined toxicity between nanomaterials with different activities. Studying the environmental behavior and ecotoxicity of nanomaterials and evaluating the ecological risks and potential hazards that nanomaterials may bring are the prerequisites for scientific and rational development, design and use of nanomaterials, and they are also of great significance for the healthy and sustainable development of nanotechnology and ecological protection. Establishing a complete ecological risk assessment procedure for nanomaterials is of great significance to solve the problem of nanomaterial pollution and is also an important direction for future nanomaterial ecotoxicology research.

## Figures and Tables

**Table 1:** Effect of NPs on microbial of different environments.

Type of NPs	Subject	Effects of Exposure to the Microbial	Dosage	Duration	Evaluation Method	Ref.
Ag NPs	wastewater treatment plants	The SBR microbial community composition shifted immediately upon exposure to Ag^+^ but recovered quickly, while the Ag NP-treated communities shifted and recovered more slowly, with the longest lasting effect produced by GA-Ag NPs.	0.2 and 2 ppm	94 d	16S rDNA, SBR treatment efficiency	[47]
	natural water bodies	A broad range of microbial endpoints as well as rates of litter decomposition were strongly affected.	0.05 and 0.5 uM	25 d	Automated ribosomal intergenic spacer analysis (ARISA), Leaf Mass Loss	[80]
	soil	Notable impact on microbial functional and genomic diversity. Emergence of a silver tolerant bacterium was observed at Ag NP concentrations of 49–287 mg kg^−1^ after 14–28 days of incubation	49 to 1815 mg kg^−1^	28 d	heterotrophic plate counting, microbial respiration, organic matter decomposition, soil enzyme activity, biological nitrification, community level physiological profifiling (CLPP), Ion TorrentDNA sequencing and denaturinggradient gel electrophoresis (DGGE)	[105]
ZnO NPs	wastewater treatment plants	Results show that the species richness in the EBPR system was reduced under the condition of ZnO NPs with high concentration.	2–6 mg/L	43 d	High-throughput sequencing, P-removal process	[51]
	natural water bodies	A significant decrease of the microbial biomass and enzyme activities was observed in the ZnO NP exposure microcosms.	100 mg L^−1^	45 d	Extracellular enzyme activities, High-throughput pyrosequencing	[79]
	soil	Nano-ZnO reduced both microbial biomass (as indicated by declines in both SIR and DNA) and diversity (by T-RFLP) and altered the composition of the soil bacterial community.	0.05, 0.1, and 0.5 mg g^−1^	60 d	substrate induced respiration (SIR) and total extractable soil DNA, terminal restriction fragment length polymorphism (T-RFLP) analysis	[31]
CuO NPs	wastewater treatment plants	NPs performed immediate and durable toxicity on Anammox. The nitrogen removal efficiency decreased, the Anammox rate decreased and the relative abundance of AAOB decreased	1 g L^−1^	63 d	batch experiments, High-throughput pyrosequencing and phylogenetic assignment	[55]
Fe_3_O_4_ NPs	wastewater treatment plants	Fe_3_O_4_ NPs led to the toxioity to activated sludge and destroyed the integrity of microbial cytomembrane. Fe_3_O_4_ NPs could obviously affect the microbial richness and diversity of activated sludge.	5–60 mg/L	57 d	the dichlorodihydroflfluorescein (DCF) assay method, a LDH kit, the high-throughput sequencing	[62]
TiO_2_ NPs	wastewater treatment plants	50 mg/L TiO_2_ NPs was observed to significantly decrease total nitrogen (TN) removal efficiency after long-term exposure (70 days), and obviously reduced the diversity of microbial community in activated sludge. The abundance of nitrifying bacteria, especially ammonia-oxidizing bacteria, was highly decreased	0.15–0.50 mg/L	70 d	total nitrogen (TN) removal efficiency, fluorescence in situ hybridization analysis	[65]
	soil	The biomass of total phospho lipid fatty acid (PLFA), Gram positive, Gram negative bacteria, fungi, actinomyctetes and anaerobes were found to be increased up to dose of 80 mg TiO_2_ NPs kg^−1^ soil, but, significantly declined at 100 mg TiO_2_ NPs kg^−1^ soil dose	5, 10, 20, 40, 80, 100 mg kg^−1^	45 d	fluorescein diacetate (FDA) hydrolyzing capacity, phospholipid fatty acid (PLFA) analysis	[109]
CeO_2_ NPs	wastewater treatment plants	The presence of CeO_2_ NPs had obvious effect on the microbial richness and diversity of activated sludge. High CeO_2_ NPs concentration could result in the biotoxicity to activated sludge	5–60 mg/L	290 d	the dichlorodihydroflfluorescein (DCF)	[66]
	soil	CeO_2_ NPs were observed to hinder thermogenic metabolism, reduce numbers of soil Azotobacter, P-solubilizing and K-solubilizing bacteria and inhibit enzymatic activities.	1 mg g^−1^	30 d	thermal metabolism, the abundance of functional bacteria and enzymatic activity.	[112]

## Data Availability

Not applicable.

## References

[1] Mousavi S.Z., Nafisi S., Maibach H.I. (2017). Fullerene nanoparticle in dermatological and cosmetic applications. Nanomed. Nanotechnol. Biol. Med..

[2] Vijayakumar S., Vaseeharan B., Malaikozhundan B., Gobi N., Ravichandran S., Karthi S., Ashokkumar B., Sivakumar N. (2017). A novel antimicrobial therapy for the control of Aeromonas hydrophila infection in aquaculture using marine polysaccharide coated gold nanoparticle. Microb. Pathog..

[3] Adams J., Wright M., Wagner H., Valiente J., Britt D., Anderson A. (2017). Cu from dissolution of CuO nanoparticles signals changes in root morphology. Plant Physiol. Biochem..

[4] Athanassiou C.G., Kavallieratos N.G., Benelli G., Losic D., Rani P.U., Desneux N. (2018). Nanoparticles for pest control: Current status and future perspectives. J. Pest. Sci..

[5] Elmer W., Ma C., White J. (2018). Nanoparticles for plant disease management. Curr. Opin. Environ. Sci. Health.

[6] Mitrano D.M., Lombi E., Dasilva Y.A.R., Nowack B. (2016). Unraveling the Complexity in the Aging of Nanoenhanced Textiles: A Comprehensive Sequential Study on the Effects of Sunlight and Washing on Silver Nanoparticles. Environ. Sci. Technol..

[7] Namdari M., Eatemadi A., Soleimaninejad M., Hammed A.T. (2017). A brief review on the application of nanoparticle enclosed herbal medicine for the treatment of infective endocarditis. Biomed. Pharmacother..

[8] Yalcinkaya F., Komarek M. (2019). Polyvinyl Butyral (PVB) Nanofiber/Nanoparticle-Covered Yarns for Antibacterial Textile Surfaces. Int. J. Mol. Sci..

[9] Rai P.K., Kumar V., Lee S., Raza N., Kim K., Ok Y.S., Tsang D.C.W. (2018). Nanoparticle-plant interaction: Implications in energy, environment, and agriculture. Environ. Int..

[10] Tavakoli M., Malakooti M.H., Paisana H., Ohm Y., Marques D.G., Lopes P.A., Piedade A.P., de Almeida A.T., Majidi C. (2018). EGaIn-Assisted Room-Temperature Sintering of Silver Nanoparticles for Stretchable, Inkjet-Printed, Thin-Film Electronics. Adv. Mater..

[11] Godwin H.A., Chopra K., Bradley K.A., Cohen Y., Harthorn B.H., Hoek E.M.V., Holden P., Keller A.A., Lenihan H.S., Nisbet R.M. (2009). The University of California Center for the Environmental Implications of Nanotechnology. Environ. Sci. Technol..

[12] Donia D.T., Carbone M. (2019). Fate of the nanoparticles in environmental cycles. Int. J. Environ. Sci. Technol..

[13] Colvin V.L. (2003). The potential environmental impact of engineered nanomaterials. Nat. Biotechnol..

[14] Klaine S.J., Alvarez P.J.J., Batley G.E., Fernandes T.F., Handy R.D., Lyon D.Y., Mahendra S., McLaughlin M.J., Lead J.R. (2008). Nanomaterials in the environment: Behavior, fate, bioavailability, and effects. Environ. Toxicol. Chem..

[15] Grieger K.D., Hansen S.F., Baun A. (2009). The known unknowns of nanomaterials: Describing and characterizing uncertainty within environmental, health and safety risks. Nanotoxicology.

[16] Long T.C., Saleh N., Tilton R.D., Lowry G.V., Veronesi B. (2006). Titanium dioxide (P25) produces reactive oxygen species in immortalized brain microglia (BV2): Implications for nanoparticle neurotoxicity. Environ. Sci. Technol..

[17] Woerle-Knirsch J.M., Kern K., Schleh C., Adelhelm C., Feldmann C., Krug H.F. (2007). Nanoparticulate vanadium oxide potentiated vanadium toxicity in human lung cells. Environ. Sci. Technol..

[18] Jiang W., Kim B.Y.S., Rutka J.T., Chan W.C.W. (2008). Nanoparticle-mediated cellular response is size-dependent. Nat. Nanotechnol..

[19] Baek Y., An Y. (2011). Microbial toxicity of metal oxide nanoparticles (CuO, NiO, ZnO, and Sb_2_O_3_) to *Escherichia coli*, *Bacillus subtilis*, and *Streptococcus aureus*. Sci. Total Environ..

[20] Manzo S., Miglietta M.L., Rametta G., Buono S., di Francia G. (2013). Toxic effects of ZnO nanoparticles towards marine algae *Dunaliella tertiolecta*. Sci. Total Environ..

[21] Mesak C., Dos Reis Sampaio D.M., de Oliveira Ferreira R., de Oliveira Mendes B., de Lima Rodrigues A.S., Malafaia G. (2018). The effects of predicted environmentally relevant concentrations of ZnO nanoparticles on the behavior of *Gallus gallus domesticus* (*Phasianidae*) chicks. Environ. Pollut..

[22] Mouneyrac C., Buffet P., Poirier L., Zalouk-Vergnoux A., Guibbolini M., Faverney C.R., Gilliland D., Berhanu D., Dybowska A., Chatel A. (2014). Fate and effects of metal-based nanoparticles in two marine invertebrates, the bivalve mollusc *Scrobicularia plana* and the annelid polychaete *Hediste diversicolor*. Environ. Sci. Pollut. Res. Int..

[23] Servin A.D., Castillo-Michel H., Hernandez-Viezcas J.A., Diaz B.C., Peralta-Videa J.R., Gardea-Torresdey J.L. (2012). Synchrotron Micro-XRE and Micro-XANES Confirmation of the Uptake and Translocation of TiO_2_ Nanoparticles in Cucumber (*Cucumis sativus*) Plants. Environ. Sci. Technol..

[24] Kumari M., Khan S.S., Pakrashi S., Mukherjee A., Chandrasekaran N. (2011). Cytogenetic and genotoxic effects of zinc oxide nanoparticles on root cells of *Allium cepa*. J. Hazard. Mater..

[25] Sharma V., Anderson D., Dhawan A. (2011). Zinc Oxide Nanoparticles Induce Oxidative Stress and Genotoxicity in Human Liver Cells (HepG2). J. Biomed. Nanotechnol..

[26] Sharma V., Anderson D., Dhawan A. (2012). Zinc oxide nanoparticles induce oxidative DNA damage and ROS-triggered mitochondria mediated apoptosis in human liver cells (HepG2). Apoptosis.

[27] Sharma V., Shukla R.K., Saxena N., Parmar D., Das M., Dhawan A. (2009). DNA damaging potential of zinc oxide nanoparticles in human epidermal cells. Toxicol. Lett..

[28] Mihai C., Chrisler W.B., Xie Y., Hu D., Szymanski C.J., Tolic A., Klein J.A., Smith J.N., Tarasevich B.J., Orr G. (2015). Intracellular accumulation dynamics and fate of zinc ions in alveolar epithelial cells exposed to airborne ZnO nanoparticles at the air-liquid interface. Nanotoxicology.

[29] Moore M.N. (2006). Do nanoparticles present ecotoxicological risks for the health of the aquatic environment?. Environ. Int..

[30] Nogueira V., Lopes I., Rocha-Santos T., Santos A.L., Rasteiro G.M., Antunes F., Goncalves F., Soares A.M.V.M., Cunha A., Almeida A. (2012). Impact of organic and inorganic nanomaterials in the soil microbial community structure. Sci. Total Environ..

[31] Ge Y., Schimel J.P., Holden P.A. (2011). Evidence for Negative Effects of TiO_2_ and ZnO Nanoparticles on Soil Bacterial Communities. Environ. Sci. Technol..

[32] Frenk S., Ben-Moshe T., Dror I., Berkowitz B., Minz D. (2003). Effect of Metal Oxide Nanoparticles on Microbial Community Structure and Function in Two Different Soil Types. PLoS ONE.

[33] Kaegi R., Ulrich A., Sinnet B., Vonbank R., Wichser A., Zuleeg S., Simmler H., Brunner S., Vonmont H., Burkhardt M. (2008). Synthetic TiO_2_ nanoparticle emission from exterior facades into the aquatic environment. Environ. Pollut..

[34] Mitrano D.M., Rimmele E., Wichser A., Erni R., Height M., Nowack B. (2014). Presence of Nanoparticles in Wash Water from Conventional Silver and Nano-silver Textiles. ACS Nano.

[35] Tang S.C.N., Lo I.M.C. (2013). Magnetic nanoparticles: Essential factors for sustainable environmental applications. Water Res..

[36] Lee S., Laldawngliana C., Tiwari D. (2012). Iron oxide nano-particles-immobilized-sand material in the treatment of Cu(II), Cd(II) and Pb(II) contaminated waste waters. Chem. Eng. J..

[37] Chai L., Wang Y., Zhao N., Yang W., You X. (2013). Sulfate-doped Fe_3_O_4_/Al_2_O_3_ nanoparticles as a novel adsorbent for fluoride removal from drinking water. Water Res..

[38] Marin S., Vlasceanu G.M., Tiplea R.E., Bucur I.R., Lemnaru M., Marin M.M., Grumezescu A.M. (2015). Applications and Toxicity of Silver Nanoparticles: A Recent Review. Curr. Top. Med. Chem..

[39] Weinberg H., Galyean A., Leopold M. (2011). Evaluating engineered nanoparticles in natural waters. Trac.-Trend. Anal. Chem..

[40] Wiesner M.R., Lowry G.V., Alvarez P., Dionysiou D., Biswas P. (2006). Assessing the risks of manufactured nanomaterials. Environ. Sci. Technol..

[41] Hartmann R.R., Kono J., Portnoi M.E. (2014). Terahertz science and technology of carbon nanomaterials. Nanotechnology.

[42] Vaseashta A., Vaclavikova M., Vaseashta S., Gallios G., Roy P., Pummakarnchana O. (2007). Nanostructures in environmental pollution detection, monitoring, and remediation. Sci. Technol. Adv. Mater..

[43] Ganesh R., Smeraldi J., Hosseini T., Khatib L., Olson B.H., Rosso D. (2010). Evaluation of Nanocopper Removal and Toxicity in Municipal Wastewaters. Environ. Sci. Technol..

[44] Zhang Z., Xu J., Shi Z., Cheng Y., Ji Z., Deng R., Jin R. (2017). Combined impacts of nanoparticles on anammox granules and the roles of EDTA and S2^−^ in attenuation. J. Hazard. Mater..

[45] Button M., Auvinen H., van Koetsem F., Hosseinkhani B., Rousseau D., Weber K.P., Laing G.D. (2016). Susceptibility of constructed wetland microbial communities to silver nanoparticles: A microcosm study. Ecol. Eng..

[46] Xu Q., Li S., Wan Y., Wang S., Ma B., She Z., Guo L., Gao M., Zhao Y., Jin C. (2017). Impacts of silver nanoparticles on performance and microbial community and enzymatic activity of a sequencing batch reactor. J. Environ. Manag..

[47] Gwin C.A., Lefevre E., Alito C.L., Gunscha C.K. (2018). Microbial community response to silver nanoparticles and Ag+ in nitrifying activated sludge revealed by ion semiconductor sequencing. Sci. Total Environ..

[48] Cao C., Huang J., Yan C., Liu J., Hu Q., Guan W. (2018). Shifts of system performance and microbial community structure in a constructed wetland after exposing silver nanoparticles. Chemosphere.

[49] Yang Y., Zhang C., Hu Z. (2013). Impact of metallic and metal oxide nanoparticles on wastewater treatment and anaerobic digestion. Environ. Sci.-Proc. Imp..

[50] Turan N.B., Erkan H.S., Engin G.O., Bilgili M.S. (2019). Nanoparticles in the aquatic environment: Usage, properties, transformation and toxicity-A review. Process. Saf. Environ..

[51] Hu Z., Lu X., Sun P., Hu Z., Wang R., Lou C., Han J. (2017). Understanding the performance of microbial community induced by ZnO nanoparticles in enhanced biological phosphorus removal system and its recoverability. Bioresour. Technol..

[52] Wu Q., Huang K., Sun H., Ren H., Zhang X., Ye L. (2018). Comparison of the impacts of zinc ions and zinc nanoparticles on nitrifying microbial community. J. Hazard. Mater..

[53] Zhang X., Zhang N., Fu H., Chen T., Liu S., Zheng S., Zhang J. (2017). Effect of zinc oxide nanoparticles on nitrogen removal, microbial activity and microbial community of CANON process in a membrane bioreactor. Bioresour. Technol..

[54] Chen Y., Yang Z., Zhang Y., Xiang Y., Xu R., Jia M., Cao J., Xiong W. (2020). Effects of different conductive nanomaterials on anaerobic digestion process and microbial community of sludge. Bioresour. Technol..

[55] Zhang X., Zhou Y., Xu T., Zheng K., Zhang R., Peng Z., Zhang H. (2018). Toxic effects of CuO, ZnO and TiO_2_ nanoparticles in environmental concentration on the nitrogen removal, microbial activity and community of Anammox process. Chem. Eng. J..

[56] Cao J.S., Elliott D., Zhang W.X. (2005). Perchlorate reduction by nanoscale iron particles. J. Nanopart. Res..

[57] Mueller N.C., Braun J., Bruns J., Cernik M., Rissing P., Rickerby D., Nowack B. (2012). Application of nanoscale zero valent iron (NZVI) for groundwater remediation in Europe. Environ. Sci. Pollut. Res..

[58] Zhou Z., Dai C., Zhou X., Zhao J., Zhang Y. (2015). The Removal of Antimony by Novel NZVI-Zeolite: The Role of Iron Transformation. Water Air Soil Poll..

[59] Yang Y., Guo J., Hu Z. (2013). Impact of nano zero valent iron (NZVI) on methanogenic activity and population dynamics in anaerobic digestion. Water Res..

[60] Yu B., Huang X., Zhang D., Lou Z., Yuan H., Zhu N. (2016). Response of sludge fermentation liquid and microbial community to nano zero-valent iron exposure in a mesophilic anaerobic digestion system. RSC Adv..

[61] Pan X., Lv N., Li C., Ning J., Wang T., Wang R., Zhou M., Zhu G. (2019). Impact of nano zero valent iron on tetracycline degradation and microbial community succession during anaerobic digestion. Chem. Eng. J..

[62] Ma B., Wang S., Li Z., Gao M., Li S., Guo L., She Z., Zhao Y., Zheng D., Jin C. (2017). Magnetic Fe_3_O_4_ nanoparticles induced effects on performance and microbial community of activated sludge from a sequencing batch reactor under long-term exposure. Bioresour. Technol..

[63] Zhang Z., Cheng Y., Bai Y., Xu L., Xu J., Shi Z., Zhang Q., Jin R. (2018). Enhanced effects of maghemite nanoparticles on the flocculent sludge wasted from a high-rate anammox reactor: Performance, microbial community and sludge characteristics. Bioresour. Technol..

[64] Li Z., Wang X., Ma B., Wang S., Zheng D., She Z., Guo L., Zhao Y., Xu Q., Jin C. (2017). Long-term impacts of titanium dioxide nanoparticles (TiO_2_ NPs) on performance and microbial community of activated sludge. Bioresour. Technol..

[65] Zheng X., Chen Y., Wu R. (2011). Long-Term Effects of Titanium Dioxide Nanoparticles on Nitrogen and Phosphorus Removal from Wastewater and Bacterial Community Shift in Activated Sludge. Environ. Sci. Technol..

[66] Wang S., Gao M., Li Z., She Z., Wu J., Zheng D., Guo L., Zhao Y., Gao F., Wang X. (2016). Performance evaluation, microbial enzymatic activity and microbial community of a sequencing batch reactor under long-term exposure to cerium dioxide nanoparticles. Bioresour. Technol..

[67] Wang S., Li Z., Gao M., She Z., Guo L., Zheng D., Zhao Y., Ma B., Gao F., Wang X. (2017). Long-term effects of nickel oxide nanoparticles on performance, microbial enzymatic activity, and microbial community of a sequencing batch reactor. Chemosphere.

[68] Chen L., Wang Y., Cao C., Liu C., Zhu L. (2017). Response of anaerobic membrane bioreactor to the presence of nano-Bi2WO6: Reactor performance, supernatant characteristics, and microbial community. Environ. Sci. Pollut. Res..

[69] Li S., Gao S., Wang S., Ma B., Guo L., Li Z., Xu Q., She Z., Gao M., Zhao Y. (2017). Performance evaluation and microbial community shift of a sequencing batch reactor under silica nanoparticles stress. Bioresour. Technol..

[70] Zhang X., Zhou Y., Yu B., Zhang N., Wang L., Fu H., Zhang J. (2017). Effect of copper oxide nanoparticles on the ammonia removal and microbial community of partial nitrification process. Chem. Eng. J..

[71] Reijnders L. (2009). The release of TiO_2_ and SiO_2_ nanoparticles from nanocomposites. Polym. Degrad. Stabil..

[72] Crump B.C., Hopkinson C.S., Sogin M.L., Hobbie J.E. (2004). Microbial biogeography along an estuarine salinity gradient: Combined influences of bacterial growth and residence time. Appl. Environ. Microb..

[73] Egger M., Rasigraf O., Sapart C.J., Jilbert T., Jetten M.S.M., Röckmann T., van der Veen C., Bândă N., Kartal B., Ettwig K.F. (2015). Iron-Mediated Anaerobic Oxidation of Methane in Brackish Coastal Sediments. Environ. Sci. Technol..

[74] Bauer J.E., Cai W., Raymond P.A., Bianchi T.S., Hopkinson C.S., Regnier P.A.G. (2013). The changing carbon cycle of the coastal ocean. Nature.

[75] Besaury L., Marty F., Buquet S., Mesnage V., Muyzer G., Quillet L. (2013). Culture-Dependent and Independent Studies of Microbial Diversity in Highly Copper-Contaminated Chilean Marine Sediments. Microb. Ecol..

[76] Ribeiro H., Mucha A.P., Almeida C.M.R., Bordalo A.A. (2013). Bacterial community response to petroleum contamination and nutrient addition in sediments from a temperate salt marsh. Sci. Total Environ..

[77] Londono N., Donovan A.R., Shi H., Geisler M., Liang Y. (2017). Impact of TiO_2_ and ZnO nanoparticles on an aquatic microbial community: Effect at environmentally relevant concentrations. Nanotoxicology.

[78] Bao S., Wang H., Zhang W., Xie Z., Fang T. (2016). An investigation into the effects of silver nanoparticles on natural microbial communities in two freshwater sediments. Environ. Pollut..

[79] Du J., Zhang Y., Cui M., Yang J., Lin Z., Zhang H. (2017). Evidence for negative effects of ZnO nanoparticles on leaf litter decomposition in freshwater ecosystems. Environ. Sci.-Nano..

[80] Tlili A., Jabiol J., Behra R., Gil-Allué C., Gessner M.O. (2017). Chronic Exposure Effects of Silver Nanoparticles on Stream Microbial Decomposer Communities and Ecosystem Functions. Environ. Sci. Technol..

[81] Miao L., Wang C., Hou J., Wang P., Ao Y., Li Y., Yao Y., Lv B., Yang Y., You G. (2017). Influence of silver nanoparticles on benthic oxygen consumption of microbial communities in freshwater sediments determined by microelectrodes. Environ. Pollut..

[82] Zhang Z., Zheng M., Chen B., Pan Y., Yang Z., Qian H. (2020). Nano-Sized Polystyrene at 1 mg/L Concentrations Does Not Show Strong Disturbance on the Freshwater Microbial Community. Bull. Environ. Contam. Toxicol..

[83] Ukisu Y., Miyadera T. (2003). Hydrogen-transfer hydrodechlorination of polychlorinated dibenzo-p-dioxins and dibenzofurans catalyzed by supported palladium catalysts. Appl. Catal. B Environ..

[84] Nuzzo A., Hosseinkhani B., Boon N., Zanaroli G., Fava F. (2017). Impact of bio-palladium nanoparticles (bio-Pd NPs) on the activity and structure of a marine microbial community. Environ. Pollut..

[85] Doiron K., Pelletier E., Lemarchand K. (2012). Impact of polymer-coated silver nanoparticles on marine microbial communities: A microcosm study. Aquat. Toxicol..

[86] Nowack B., Ranville J.F., Diamond S., Gallego-Urrea J.A., Metcalfe C., Rose J., Horne N., Koelmans A.A., Klaine S.J. (2012). Potential scenarios for nanomaterial release and subsequent alteration in the environment. Environ. Toxicol. Chem..

[87] Servin A., Elmer W., Mukherjee A., de la Torre-Roche R., Hamdi H., White J.C., Bindraban P., Dimkpa C. (2015). A review of the use of engineered nanomaterials to suppress plant disease and enhance crop yield. J. Nanopart. Res..

[88] Mishra S., Keswani C., Abhilash P.C., Fraceto L.F., Singh H.B. (2017). Integrated Approach of Agri-nanotechnology: Challenges and Future Trends. Front. Plant. Sci..

[89] Jiang D., Zeng G., Huang D., Chen M., Zhang C., Huang C., Wan J. (2018). Remediation of contaminated soils by enhanced nanoscale zero valent iron. Environ. Res..

[90] He J., Wang D., Zhou D. (2019). Transport and retention of silver nanoparticles in soil: Effects of input concentration, particle size and surface coating. Sci. Total Environ..

[91] Khot L.R., Sankaran S., Maja J.M., Ehsani R., Schuster E.W. (2012). Applications of nanomaterials in agricultural production and crop protection: A review. Crop. Prot..

[92] Boxall A.B.A., Tiede K., Chaudhry Q. (2007). Engineered nanomaterials in soils and water: How do they behave and could they pose a risk to human health?. Nanomed. UK.

[93] Nowack B., Bucheli T.D. (2007). Occurrence, behavior and effects of nanoparticles in the environment. Environ. Pollut..

[94] Navarro E., Baun A., Behra R., Hartmann N.B., Filser J., Miao A., Quigg A., Santschi P.H., Sigg L. (2008). Environmental behavior and ecotoxicity of engineered nanoparticles to algae, plants, and fungi. Ecotoxicology.

[95] Gottschalk F., Sonderer T., Scholz R.W., Nowack B. (2009). Modeled Environmental Concentrations of Engineered Nanomaterials (TiO_2_, ZnO, Ag, CNT, Fullerenes) for Different Regions. Environ. Sci. Technol..

[96] Funari V., Mantovani L., Vigliotti L., Tribaudino M., Dinelli E., Braga R. (2018). Superparamagnetic iron oxides nanoparticles from municipal solid waste incinerators. Sci. Total Environ..

[97] Yang Y., Wang J., Xiu Z., Alvarez P.J.J. (2013). Impacts of silver nanoparticles on cellular and transcriptional activity of nitrogen-cycling bacteria. Environ. Toxicol. Chem..

[98] Benoit R., Wilkinson K.J., Sauve S. (2013). Partitioning of silver and chemical speciation of free Ag in soils amended with nanoparticles. Chem. Cent. J..

[99] Moghaddasi S., Fotovat A., Khoshgoftarmanesh A.H., Karimzadeh F., Khazaei H.R., Khorassani R. (2017). Bioavailability of coated and uncoated ZnO nanoparticles to cucumber in soil with or without organic matter. Ecotox. Environ. Saf..

[100] Luo M., Huang Y., Zhu M., Tang Y., Ren T., Ren J., Wang H., Li F. (2018). Properties of different natural organic matter influence the adsorption and aggregation behavior of TiO_2_ nanoparticles. J. Saudi. Chem. Soc..

[101] el Hadri H., Louie S.M., Hackley V.A. (2018). Assessing the interactions of metal nanoparticles in soil and sediment matrices—A quantitative analytical multi-technique approach. Environ. Sci.-Nano..

[102] Read D.S., Matzke M., Gweon H.S., Newbold L.K., Heggelund L., Ortiz M.D., Lahive E., Spurgeon D., Svendsen C. (2016). Soil pH effects on the interactions between dissolved zinc, non-nano- and nano-ZnO with soil bacterial communities. Environ. Sci. Pollut. Res..

[103] Bundschuh M., Filser J., Luderwald S., Mckee M.S., Metreveli G., Schaumann G.E., Schulz R., Wagner S. (2018). Nanoparticles in the environment: Where do we come from, where do we go to?. Environ. Sci. Eur..

[104] Liu G., Zhang M., Jin Y., Fan X., Xu J., Zhu Y., Fu Z., Pan X., Qian H. (2017). The Effects of Low Concentrations of Silver Nanoparticles on Wheat Growth, Seed Quality, and Soil Microbial Communities. Water Air Soil Pollut..

[105] Samarajeewa A.D., Velicogna J.R., Princz J.I., Subasinghe R.M., Scroggins R.P., Beaudette L.A. (2017). Effect of silver nano-particles on soil microbial growth, activity and community diversity in a sandy loam soil. Environ. Pollut..

[106] Wang J., Shu K., Zhang L., Si Y. (2017). Effects of Silver Nanoparticles on Soil Microbial Communities and Bacterial Nitrification in Suburban Vegetable Soils. Pedosphere.

[107] Zhai Y., Hunting E.R., Wouters M., Peijnenburg W.J.G.M., Vijver M.G. (2016). Silver Nanoparticles, Ions, and Shape Governing Soil Microbial Functional Diversity: Nano Shapes Micro. Front. Microbiol..

[108] Kumar N., Shah V., Walker V.K. (2011). Perturbation of an arctic soil microbial community by metal nanoparticles. J. Hazard. Mater..

[109] Ge Y., Priester J.H., de Werfhorst L.C.V., Schimel J.P., Holden P.A. (2013). Potential Mechanisms and Environmental Controls of TiO_2_ Nanoparticle Effects on Soil Bacterial Communities. Environ. Sci. Technol..

[110] Bhattacharjya S., Adhikari T., Sahu A., Patra A.K. (2021). Ecotoxicological effect of TiO_2_ nano particles on different soil enzymes and microbial community. Ecotoxicology.

[111] Asadishad B., Chahal S., Akbari A., Cianciarelli V., Azodi M., Ghoshal S., Tufenkji N. (2018). Amendment of Agricultural Soil with Metal Nanoparticles: Effects on Soil Enzyme Activity and Microbial Community Composition. Environ. Sci. Technol..

[112] Chai H., Yao J., Sun J., Zhang C., Liu W., Zhu M., Ceccanti B. (2015). The Effect of Metal Oxide Nanoparticles on Functional Bacteria and Metabolic Profiles in Agricultural Soil. Bull. Environ. Contam. Toxicol..

[113] Chung H., Kim M.J., Ko K., Kim J.H., Kwon H.A., Hong I., Park N., Lee S.W., Kim W. (2015). Effects of graphene oxides on soil enzyme activity and microbial biomass. Sci. Total Environ..

[114] He F., Wang H., Chen Q., Yang B., Gao Y., Wang L. (2015). Short-Term Response of Soil Enzyme Activity and Soil Respiration to Repeated Carbon Nanotubes Exposure. Soil Sediment Contam..

[115] Bradford A., Handy R.D., Readman J.W., Atfield A., Mühling M. (2009). Impact of Silver Nanoparticle Contamination on the Genetic Diversity of Natural Bacterial Assemblages in Estuarine Sediments. Environ. Sci. Technol..

[116] Nyberg L., Turco R.F., Nies L. (2008). Assessing the Impact of Nanomaterials on Anaerobic Microbial Communities. Environ. Sci. Technol..

[117] Shah V., Belozerova I. (2009). Influence of Metal Nanoparticles on the Soil Microbial Community and Germination of Lettuce Seeds. Water Air Soil Pollut..

[118] Tong Z., Bischoff M., Nies L., Applegate B., Turco R.F. (2007). Impact of Fullerene (C60) on a Soil Microbial Community. Environ. Sci. Technol..

[119] Shrestha B., Acosta-Martinez V., Cox S.B., Green M.J., Li S., Cañas-Carrell J.E. (2013). An evaluation of the impact of multiwalled carbon nanotubes on soil microbial community structure and functioning. J. Hazard. Mater..

[120] McGee C.F., Storey S., Clipson N., Doyle E. (2017). Soil microbial community responses to contamination with silver, aluminium oxide and silicon dioxide nanoparticles. Ecotoxicology.

[121] He S., Feng Y., Ren H., Zhang Y., Gu N., Lin X. (2011). The impact of iron oxide magnetic nanoparticles on the soil bacterial community. J. Soil. Sediment..

[122] Lu M., Zhang Z., Wang J., Zhang M., Xu Y., Wu X. (2014). Interaction of Heavy Metals and Pyrene on Their Fates in Soil and Tall Fescue (*Festuca arundinacea*). Environ. Sci. Technol..

[123] Ren Z., Xu H., Wang Y., Li Y., Han S., Ren J. (2021). Combined toxicity characteristics and regulation of residual quinolone antibiotics in water environment. Chemosphere.

[124] van der Geest H.G., Greve G.D., Boivin M.E., Kraak M., van Gestel C. (2000). Mixture toxicity of copper and diazinon to larvae of the mayfly (*Ephoron virgo*) judging additivity at different effect levels. Environ. Toxicol. Chem..

[125] Zou X., Lin Z., Deng Z., Yin D., Zhang Y. (2012). The joint effects of sulfonamides and their potentiator on *Photobacterium phosphoreum*: Differences between the acute and chronic mixture toxicity mechanisms. Chemosphere.

[126] Zhao L., Ji Y., Sun P., Li R., Xiang F., Wang H., Ruiz-Martinez J., Yang Y. (2018). Effects of individual and complex ciprofloxacin, fullerene C60, and ZnO nanoparticles on sludge digestion: Methane production, metabolism, and microbial community. Bioresour. Technol..

